# Animal Vaccination and Vaccinal Syphilis

**Published:** 1870-01

**Authors:** 


### Animal Vaccination and Vaccinal Syphilis.

M. Latour, in the *Union Medicale,* speaks of animal vaccination and vaccinal syphilis as follows: 1. The degeneration of the Jennerian virus is far from being proven. 2. There does not exist a single example of vaccinal syphilis, properly so called. 3. The excessively rare cases of syphilis inoculated by vaccination are explicable by conditions which completely exonerate vaccine virus from all injurious mixture. 4. A large number of pretended examples of syphilis following vaccination justify the most serious doubts as to the accuracy of the diagnosis. 5. Animal vaccination, simply as another cure of lymph, is deserving of encouragement, although it possesses no real or sensible advantage over vaccination from arm to arm.—*Med. Record.*

